# Conformation Effects of CpG Methylation on Single-Stranded DNA Oligonucleotides: Analysis of the Opioid Peptide Dynorphin-Coding Sequences

**DOI:** 10.1371/journal.pone.0039605

**Published:** 2012-06-29

**Authors:** Malik Mumtaz Taqi, Sebastian K. T. S. Wärmländer, Olga Yamskova, Fatemeh Madani, Igor Bazov, Jinghui Luo, Roman Zubarev, Dineke Verbeek, Astrid Gräslund, Georgy Bakalkin

**Affiliations:** 1 Department of Pharmaceutical Biosciences, Uppsala University, Uppsala, Sweden; 2 Department of Biochemistry and Biophysics, Arrhenius Laboratories for Natural Sciences, Stockholm University, Stockholm, Sweden; 3 Department of Medical Biochemistry and Biophysics, Karolinska Institute, Stockholm, Sweden; 4 Department of Genetics, University Medical Center Groningen, University of Groningen, Groningen, The Netherlands; Consejo Superior de Investigaciones Cientificas, Spain

## Abstract

Single-stranded DNA (ssDNA) is characterized by high conformational flexibility that allows these molecules to adopt a variety of conformations. Here we used native polyacrylamide gel electrophoresis (PAGE), circular dichroism (CD) spectroscopy and nuclear magnetic resonance (NMR) spectroscopy to show that cytosine methylation at CpG sites affects the conformational flexibility of short ssDNA molecules. The CpG containing 37-nucleotide *PDYN* (prodynorphin) fragments were used as model molecules. The presence of secondary DNA structures was evident from differences in oligonucleotide mobilities on PAGE, from CD spectra, and from formation of A-T, G-C, and non-canonical G-T base pairs observed by NMR spectroscopy. The oligonucleotides displayed secondary structures at 4°C, and some also at 37°C. Methylation at CpG sites prompted sequence-dependent formation of novel conformations, or shifted the equilibrium between different existing ssDNA conformations. The effects of methylation on gel mobility and base pairing were comparable in strength to the effects induced by point mutations in the DNA sequences. The conformational effects of methylation may be relevant for epigenetic regulatory events in a chromatin context, including DNA-protein or DNA-DNA recognition in the course of gene transcription, and DNA replication and recombination when double-stranded DNA is unwinded to ssDNA.

## Introduction

In the cell nucleus, DNA may be present in single-stranded form as an intermediate in gene transcription, and also during DNA replication, repair and recombination [1]–[5]. During these processes helicases unwind double-stranded DNA to single-stranded DNA (ssDNA), which serves as a template for RNA and DNA polymerases [6], and which may be stabilized by replication protein A involved in DNA replication and repair [4]. ssDNA is characterized by high conformational flexibility, allowing a variety of conformations and formation of non-canonical secondary DNA structures such as cruciforms, G-quadruplexes and triplexes. These structures are suggested to have regulatory roles in gene transcription [7], [8], DNA replication [9], and recombination [10], [11], and may also be involved in mutagenesis [8], [12], [13]. The conformational polymorphism of ssDNA molecules is sequence-dependent, as demonstrated by several methods including polyacrylamide gel electrophoresis (PAGE) [14]–[17].

Cytosine methylation at CpG and non-CpG sites is a covalent DNA modification that plays an essential role in controlling gene transcription by turning off a specific gene or by inactivating an entire X chromosome [18]–[21]. The epigenetic regulatory functions of DNA methylation are becoming increasingly clear, whereas the mechanisms of methylation-dependent gene regulation are less well understood. Cytosine methylation may regulate gene transcription by affecting the interaction of DNA with sequence-specific transcription factors and methyl-CpG-binding domain proteins, and nucleosomes assembled with non-methylated DNA are less stable than those with methylated DNA [22], [23]. Early studies demonstrated that cytosine methylation causes slight structural alterations in the B-DNA double-helix [24]–[26], changing its mechanical properties [27] and making it more prone to adopt a Z-conformation [26]. Methylated cytosine residues can be hydrated via the formation of C–H···O interactions, which constitute a structural factor in the recognition of methylated cytosine by polar residues in DNA-binding proteins [24]. Nuclear magnetic resonance (NMR) analysis has demonstrated that CpG methylation reduces the dynamics of the DNA phosphate-sugar backbone [28], while molecular dynamics simulations have suggested that methyl groups decrease DNA flexibility due to steric hindrance and hydrophobicity [24], [29]. This DNA-bending flexibility - affected by methylation - may be a critical factor in formation of nucleosomes [30], [31]. Depending on the level of methylation and the sequence context, methylation may either inhibit or facilitate DNA strand separation [27]. However, to the best of our knowledge, previous studies of cytosine methylation have been focused on double-stranded DNA.

**Figure 1:**
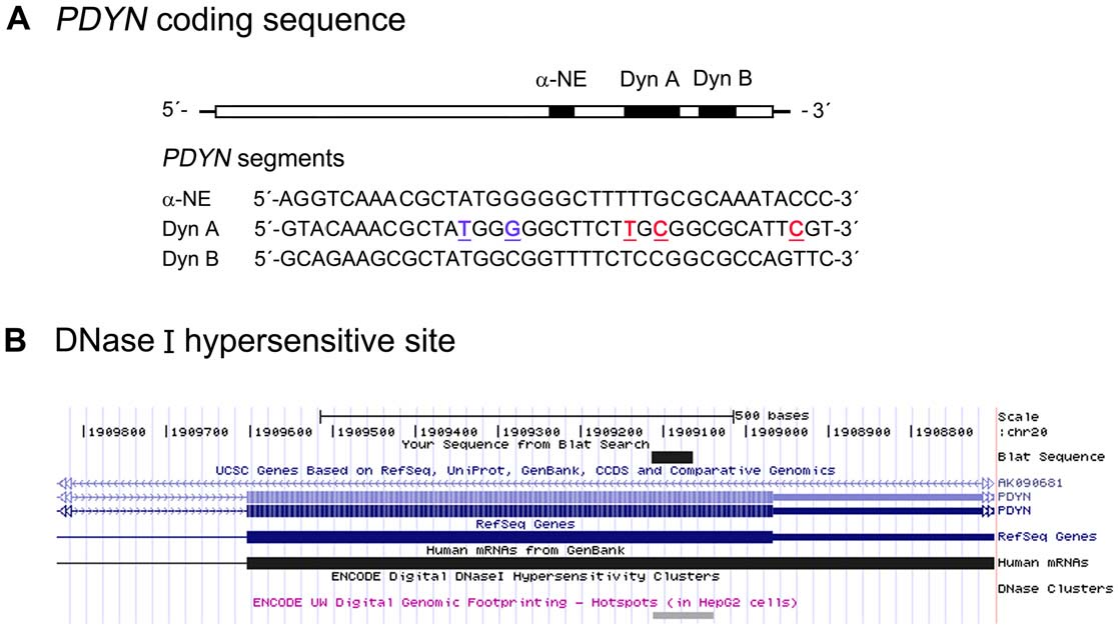
The *PDYN*-coding sequence, which gives rise to the opioid peptides α-NE, Dyn A, Dyn B. (**A**) The oligonucleotides analyzed in this study correspond to *PDYN* fragments with α-NE-, Dyn A- and Dyn B-coding sequences. Three pathogenic mutations causing the human neurodegenerative disorder SCA23 are shown in red, and two “non-natural” point mutations in blue. (**B**) **DNase hypersensitive site in the human** ***PDYN*** **-coding region.** Image was taken from the UCSC Genome Browser on Human 2006 (NCBI36/hg18) Assembly with the Dyn A-coding sequence used for the search; the region of DNase hypersensitivity overlaps with the Dyn A-coding sequence in HepG2 cells.

In this study, we evaluated the effects of CpG methylation on the conformational flexibility of short ssDNA molecules. Using native PAGE analysis and NMR and circular dichroism (CD) spectroscopy, we analyzed a set of 37-mer ssDNA oligonucleotides with two or four CpG sites in each molecule. Effects of cytosine methylation of one or two of these CpG sites were examined. Because DNA secondary structures typically are most stable at low temperatures, the experiments were performed at 4°C, and for comparison at 37°C where non-canonical DNA secondary structures usually have melted [15], [32]–[34]. Fragments of exon 4 of the human prodynorphin (*PDYN*) gene were used as model molecules because they a) contain CpG sites; and b) may be involved in DNA metabolic processes due to their potential ability to form ssDNA in the cell (Fig. 1A). These *PDYN* segments demonstrated DNase I hypersensitivity (Fig. 1B) suggesting their presence in single-stranded form in chromatin context. Furthermore, the sequences derive from a 51-nucleotide mutational “hot spot” (Fig. 1A, Table 1), containing seven mutations known to cause the human dominant neurodegenerative disorder spinocerebellar ataxia 23 (SCA23) ([35] and manuscript in preparation). A high density of these mutations along with the fact that each mutation eliminates or creates a CpG site suggests a role of methylation-dependent mutagenesis in this segment. In our analysis, the magnitudes of the conformational perturbations introduced by CpG methylation were compared to those induced by three human pathogenic missense mutations [35] and two “artificial” mutations (Fig. 1, Table 1).

**Table 1:** Oligonucleotides used in the study.

Oligonucleotide	Sequence
α-NE	5′-AGGTCAAA**CG**CTATGGGGGCTTTTTG**CG**CAAATACCC-3′
α-NE^5m^C_1_	5′-AGGTCAAA**^5m^CG**CTATGGGGGCTTTTTG**CG**CAAATACCC-3′
α-NE (AS)	5′-GGGTATTTG**CG**CAAAAAGCCCCCATAG**CG**TTTGACCT-3′
Dyn A	5′-GTACAAA**CG**CTATGGGGGCTTCTTG**CG**G**CG**CATT**CG**T-3′
Dyn A^5m^C_1_	5′-GTACAAA**^5m^CG**CTATGGGGGCTTCTTG**CG**G**CG**CATT**CG**T-3′
Dyn A^5m^C_2_	5′-GTACAAA**CG**CTATGGGGGCTTCTTG**^5m^CG**G**CG**CATT**CG**T-3′
Dyn A^5m^C_1,2_	5′-GTACAAA**^5m^CG**CTATGGGGGCTTCTTG**^5m^CG**G**CG**CATT**CG**T-3′
Dyn A^5m^C_1,3_	5′-GTACAAA**^5m^CG**CTATGGGGGCTTCTTG**CG**G**^5m^CG**CATT**CG**T-3′
Dyn A M_1_	5′-GTACAAA**CG**CTATGGGGGCTTCT***C***G**CG**G**CG**CATT**CG**T-3′
Dyn A M_2_	5′-GTACAAA**CG**CTATGGGGGCTTCTTG***T***GG**CG**CATT**CG**T-3′
Dyn A M_3_	5′-GTACAAA**CG**CTATGGGGGCTTCTTG**CG**G**CG**CATT***T***GT-3′
Dyn A M_4_	5′-GTACAAA**CG**CTATGG***C***GGCTTCTTG**CG**G**CG**CATT**CG**T-3′
Dyn A M_5_	5′-GTACAAA**CG**CTA***A***GGGGGCTTCTTG**CG**G**CG**CATT**CG**T-3′
Dyn A (AS)	5′-A**CG**AATG**CG**C**CG**CAAGAAGCCCCCATAG**CG**TTTGTAC-3′
Dyn B	5′-GCAGAAG**CG**CTATGG**CG**GTTTTCTC**CG**G**CG**CCAGTTC-3′
Dyn B^5m^C_1_	5′-GCAGAAG**^5m^CG**CTATGG**CG**GTTTTCTC**CG**G**CG**CCAGTTC-3′
Dyn B (AS)	5′-GAACTGG**CG**CCGGAGAAAAC**CG**CCATAG**CG**CTTCTGC-3′
Reference oligonucleotides (RO)	
26RO	5′-ATCAATGCCAAC**CG**CAGGTCCCTTAG-3′
37RO(+)	5′-GGTGATCAGGGACTTTCCGCTGGGGACTTTCCAGGAT-3′
37RO(−)	5′-GTGATCCTGGAAAGTGAATAG**CG**GAAAGTGAATGATC-3′
54RO	5′-G**CG**C**CG**CAAGAAGC**CG**CCATAG**CG**TTTGTACAGGTCCTCATGGCCCATGCTATC-3′

α-NE, Dyn A and Dyn B correspond to the α-neoendorphin-, dynorphin A- and dynorphin B-coding sequences of the human prodynorphin (*PDYN*) gene. AS, antisense oligonucleotide. ^5m^C, 5-methylcytosine. RO, reference oligonucleotide. (+), plus strand. (−), minus strand. M, mutations are shown in bold italic underlined letters. CpG dinucleotides are shown in bold letters. Dyn A M_1_–_3_ are oligonucleotides with human pathogenic SCA 23 mutations [35]. Dyn A M_4,5_ are oligonucleotides with nonsense and silent mutations.

## Methods

### Oligonucleotide Synthesis, Purification and Labeling

The studied oligonucleotides and their abbreviations are shown in Table 1. The oligonucleotides were synthesized and purified through high-pressure liquid chromatography (HPLC) by Eurofins MWG Operon GmbH (Ebersberg, Germany), stored at −80°C before use, and their concentrations were determined from 260 nm absorption measured using Nanodrop® (Nanodrop Technologies, Inc, USA). Additional purification of some oligonucleotides (i.e. α-NE (α- neoendorphin), α-NE^5m^C_1_ (5-methylcytosine), Dyn (dynorphin) A, Dyn A (AS (antisense)), and Dyn A^5m^C_2_) was performed using PAGE on 15% denaturating gels in 10 M urea, followed by desalting on NAP 5 and NAP 10 columns (GE Healthcare, UK). Oligonucleotide (20 ng) labeling was performed at the 5′-end using T4 polynucleotide kinase with [γ-^32^P]-ATP as a substrate, and the labeled oligonucleotides were precipitated by 66% ethanol and 1.2 M ammonium acetate in the presence of glycogen (0.01 µg/µl), followed by washing with 80% ethanol. Prior to labeling the oligonucleotides were heated for 10 min at 95°C. Native and denaturating PAGE analysis showed no differences in the behavior and purity between the HPLC purified and the double HPLC and PAGE purified oligonucleotides. The HPLC purified oligonucleotides were used in the CD and NMR experiments.

### Mass Spectrometry of Oligonucleotides

A Premier qTOF mass spectrometer (Waters, USA) using negative-mode electrospray ionization was employed to study the homogeneity of selected methylated oligonucleotide samples. The oligonucleotide samples were dissolved to a concentration of 8 g/L in 1∶1 (v/v) water-acetonitrile solution to which 3% piperidine was added to assist with deprotonation and salt adduct removal. Each mass spectrum was accumulated for 5 minutes and the data were integrated. Neutral mass deconvolution was performed using commercial software (Waters, USA). In addition, MALDI spectra provided by Eurofins MWG Operon GmbH (Ebersberg, Germany) for showing the quality of some of the oligonucleotides analyzed in this study are presented in Fig. S1 and Table S1. These experiments confirmed that the methylated oligonucleotides were homogeneous, i.e. there were no mass differences in the samples due to incomplete methylation (Table S1).

### Native and Denaturing PAGE

Native PAGE was carried out at 4°C for 5 hours or 37°C for 3 hours. Prior to the analysis, the oligonucleotides (approximately 0.5 ng per cell) were annealed at 95°C for 10 min, mixed with loading buffer, and incubated for 30 minutes or overnight at 4°C or 37°C. The loading buffer consisted of 20 mM Tris/HCl pH 7.5, 37.5% glycerol, 50 mM NaCl and 15 mM MgCl_2_. To examine effects of ion composition, NaCl and MgCl_2_ were omitted from the loading buffer, or substituted with 0.5 mM ZnCl_2_ or 100 mM KCl or 50 mM NH_4_SO_4_. The samples were analyzed on a 15% polyacrylamide (29∶1 acrylamide∶bisacrylamide) gel with 0.5× TGE (25 mM Tris-HCl, 0.2 M glycine and 1 mM EDTA) as running buffer. Four single-stranded oligonucleotides were used as reference oligomers (RO) for migration, i.e. a 26-mer (26RO), two 37-mers with (+) and (−) strands (37RO(+), 37RO(−)), and a 54-mer (54RO) (Table 1). Relative mobility (R_f_, retention factor) was calculated as the ratio of the distance migrated by an oligonucleotide to the distance migrated by the pair of 37-nt reference oligonucleotides, i.e. 37RO(+) and 37RO(−), which demonstrated virtually identical mobility on native PAGE.

For denaturing PAGE, samples were prepared in loading buffer consisting of 20 mM Tris/HCl pH 7.5, 15 mM MgCl_2_, 50 mM NaCl and 50% formamide; and run at 37°C on a 7.5 M urea, 15% polyacrylamide (19∶1 acrylamide∶bisacrylamide) gel in 1× TBE (8.9 mM Tris base, 8.9 mM boric acid and 0.2 mM EDTA).

### CD Spectroscopy

A Chirascan CD unit (Applied Photophysics, Surrey UK) was used to record CD data of the oligonucleotides dissolved in 10 mM sodium phosphate buffer at pH 7.3. A 2 mm quartz cuvette was used to hold 400 µl samples of oligonucleotides in the concentration range 20–30 µM. CD spectra between 220 and 340 nm were recorded at 4°C and at 60°C for all samples, and melting profiles between 5°C and 60°C were recorded at 275 nm at a rate of 0.2°C/min. Before the measurements all samples were annealed at 95°C for 3–5 min. The CD melting profiles were normalized using the formula (S_t_−S_60°C_)/(S_5°C_−S_60°C_), where S_t_, S_5°C_ and S_60°C_ are the 275 nm signal intensities at a given temperature, at 5°C, and at 60°C, respectively.

### NMR Spectroscopy

A Bruker Avance 500 MHz NMR spectrometer equipped with a cryogenic probehead was used to record 1D spectra at 4°C and at 37°C of unlabelled oligonucleotides dissolved in 10 mM sodium phosphate buffer at pH 7.3 (90/10 H_2_O/D_2_O). The sample concentrations were in the range 100–300 µM, with 30 µM tetramethylsilane added for reference purposes. The water signal was suppressed using the excitation sculpting method. Before the measurements all samples were annealed at 95°C for 3–5 min.

### Computer modeling

The mFold software [36] was used to predict secondary structures and folding energies for the 37 nt Dyn A and Dyn B sequences at temperatures from 5 to 55°C, using an ionic strength corresponding to 10 mM NaCl.

## Results

### PAGE Analysis

PAGE mobility depends on the molecular mass, nucleotide content, sequence, shape, and compactness, and the rigidity of the oligonucleotide molecules [37]. Oligonucleotides that form non-canonical structures such as DNA hairpins and G-quadruplexes differ in mobility on the native PAGE from unstructured oligonucleotides [38]–[44], and even single-nucleotide substitutions may induce conformational changes that can be detected with PAGE analysis [16].

**Figure 2:**
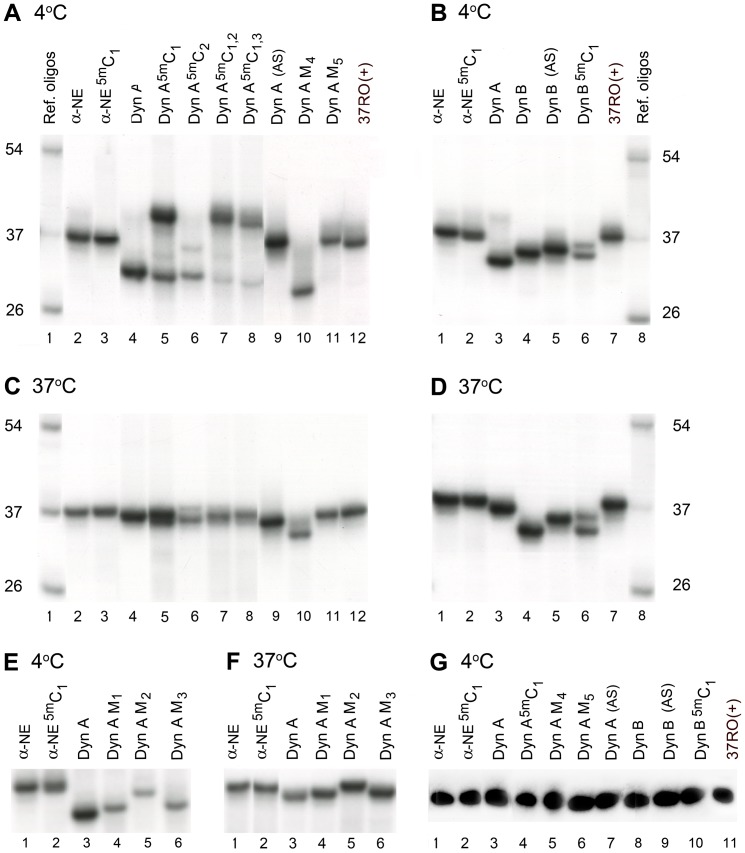
Analysis of [γ-^32^P]-labeled *PDYN*-derived oligonucleotides using PAGE (see **Table 1** **for sequences).** Reference oligonucleotides (RO) included 26-, 37- and 54-mer oligomers. Samples were preheated for 10 min at 95°C, incubated in loading buffer (20 mM Tris/HCl pH 7.5, 37.5% glycerol, 15 mM MgCl_2_ and 50 mM NaCl) at 4°C (A, B, E,) or at 37°C (C, D, F, G) for 30 minutes before loading on native (A–F) or denaturing 7.5 M urea (G) 15% polyacrylamide gel, and resolved at 4°C (A, B, E,) or at 37°C (C, D, F, G). Images shown were taken from the same gel; equal amounts of radioactive oligonucleotides were loaded.

The effects of cytosine methylation on the oligonucleotide secondary structures were analyzed by comparing the mobilities of unmethylated and methylated 37-mer oligonucleotides with the α-NE-, Dyn A- and Dyn B-coding sequences at 4°C or 37°C (Tables 1, 2; Figs. 1, 2). The α-NE, Dyn A (AS), and two 37-mer reference oligomers 37RO(+) and 37RO(−) demonstrated similar mobilities at each temperature (Fig. 2A,C, lanes 1, 2, 9, 12), suggesting similar - probably unstructured -conformations. The Dyn A oligonucleotide demonstrated a higher mobility than these four species at 4°C (Fig. 2A, lane 4), but did not differ from them in mobility at 37°C (Fig. 2C, lane 4). The Dyn B and Dyn B (AS) oligonucleotides migrated faster compared to the first four oligonucleotides at both temperatures (Fig. 2B,D, lanes 4, 5), which may be explained by differences in nucleotide sequence or content, or by acquisition of conformations stable also at 37°C.

**Table 2:** Mobility on native PAGE and number of base-pair hydrogen bonds obtained from NMR experiments for *PDYN* derived oligonucleotides.

Oligonucleotide	Relative mobility (R_f_) on PAGE^a^		No. of hydrogen-bonded imino protons from base pairs observed with NMR spectroscopy			
	4°C	37°C	4°C			37°C
			A-T	C-G	G-T or nonspecific	
α-NE	1.05	1.01	2	5	1	2 G-C
α-NE^5m^C_1_	1.05	1.01	2	4	3	2 A-T; 2 G-C
Dyn A	1.13	1.03	3	4	3	-
Dyn A^5m^C_1_	0.97	1.01	5	3	3	-
Dyn A^5m^C_2_	1.13	1.01	3	3	3	-
Dyn A^5m^C_1,2_	0.97	1.03	4	3	4	-
Dyn A^5m^C_1,3_	0.98	1.02	4	3	3	-
Dyn A M_1_	1.11	1.02	2	1	1	-
Dyn A M_2_	1.07	1.00	1	1	-	-
Dyn A M_3_	1.12	1.02	3	3	4	-
Dyn A M_4_	1.14	1.09	2	3	3	-
Dyn A M_5_	0.99	1.03	2	2	3	1 G-T
Dyn A (AS)	1.02	1.03	n/a	n/a	n/a	n/a
Dyn B	1.09	1.10	2	3	6	1 G-C
Dyn B^5m^C_1_	1.06; 1.09^b^	1.04; 1.08^b^	2	5	7	-
Dyn B (AS)	1.06	1.05	n/a	n/a	n/a	n/a

The oligonucleotide sequences and corresponding names are given in Table 1.

a , standard deviation for relative mobility values calculated using data of 2–6 experiments did not exceed 0.02.

-, no imino protons were observed.

n/a, no measurements were carried out.

b , R_f_ was calculated for dominant bands, except Dyn B^5m^C_1_, where calculation was carried out for the lower (left value) and upper (right value) bands showing similar intensity (see Fig. 2).

Methylation of the Dyn A-oligonucleotide at the first CpG site from the 5′-end (Dyn A^5m^C_1_) resulted in formation of a dominant slowly migrating conformer at 4°C, while methylation of the second CpG site (Dyn A^5m^C_2_) produced no changes in mobility (Fig. 2A, lanes 5, 6). Methylation of both the first and second sites (Dyn A^5m^C_1,2_), or the first and third sites (Dyn A^5m^C_1,3_) resulted in patterns similar to that produced by the Dyn A^5m^C_1_ oligomer (Fig. 2A, lanes 7, 8). At 37°C, the methylated Dyn A oligonucleotides did not differ in mobility from the unmethylated Dyn A oligomer (Fig. 2C, lanes 4–8). No mobility effect from methylation of the α-NE oligomer at the first CpG site was observed (Fig. 2A, lane 3). Methylation of the Dyn B oligomer at the first CpG site yielded a conformer with decreased mobility at both 4°C and 37°C (Fig. 2B,D, lane 6).

The methylation effects were compared to the effects induced by five point mutations (M) in the Dyn A oligonucleotide sequence, including the three human pathogenic mutations causing SCA23 (M_1_–M_3_), a G to C substitution in the middle of the G run (M_4_), and a T to A substitution producing an in-frame stop codon (M_5_) (Tables 1 and 2). The SCA23 mutation M_2_, but not M_1_ or M_3_, resulted in a substantial mobility decrease at 4°C (Fig. 2E, lanes 4–6), while at 37°C these three mutants showed a similar migration pattern (Fig. 2F, lanes 4–6). The Dyn A M_4_ oligonucleotide showed elevated mobility at both temperatures compared to the wild-type oligomer (Fig. 2A,C, lanes 10), while the M_5_ mutation resulted in decreased mobility at 4°C but not at 37°C (Fig. 2A, C, lanes 11).

The migration patterns on native PAGE shown in Fig. 2 did not depend on the presence or absence of mono- and divalent ions including 50 mM NaCl, 15 mM MgCl_2_, 100 mM KCl, 50 mM NH_4_SO_4_ or 0.5 mM ZnCl_2_ in the loading buffer during the pre-incubation period, nor on whether this period was 10 or 30 min, or 18 h, before loading on the gel (data not shown). All analyzed 37-mer oligonucleotides demonstrated virtually identical mobility on the denaturing PAGE (Fig. 2G).

**Figure 3:**
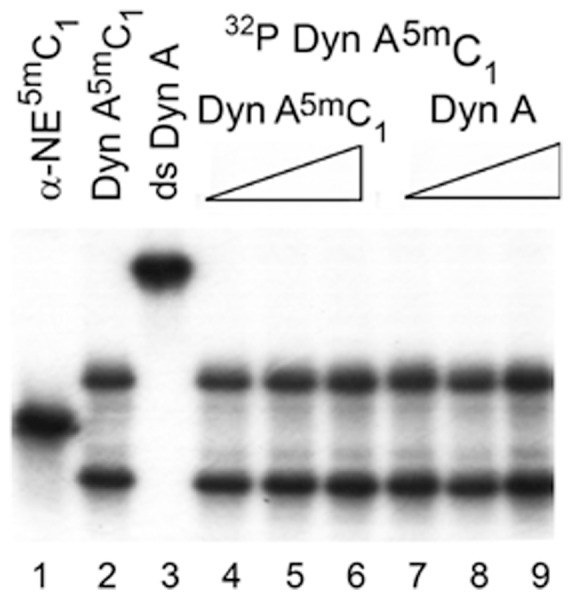
Effects of excess of unlabeled Dyn A and Dyn A^5m^C_1_ oligonucleotides on migration of [γ-^32^P]-labeled Dyn A^5m^C_1_ oligonucleotide on a native gel. Samples were preheated for 10 min at 95°C, mixed with loading buffer (20 mM Tris/HCl pH 7.5, 37.5% glycerol, 15 mM MgCl_2_ and 50 mM NaCl), incubated for 30 minutes at 4°C, and resolved on native 15% polyacrylamide gel at 4°C. Lane 1, [γ-^32^P]-labeled α-NE^5m^C_1_ oligonucleotide; lane 2, [γ-^32^P]-labeled Dyn A^5m^C_1_ oligonucleotide; lane 3, double-stranded (ds) oligonucleotide produced by preincubation of [γ-^32^P]-labeled Dyn A oligonucleotide with the corresponding antisense oligonucleotide; lanes 4 to 6 and 7 to 9, [γ-^32^P]-labeled Dyn A^5m^C_1_ oligonucleotide preincubated with 0.7, 7.0 or 700.0 ng of unlabeled Dyn A^5m^C_1_ or Dyn A oligonucleotide.

The upper band of the Dyn A^5m^C_1_ oligonucleotide (Fig. 2A, lane 5) could conceivably represent a molecular dimer. If so, the increase in oligonucleotide concentration should shift the equilibrium in favor of the dimer. Analysis of labeled Dyn A^5m^C_1_ oligonucleotides (Fig. 3, lane 2) preincubated with unlabeled Dyn A^5m^C_1_ or Dyn A oligonucleotides did not confirm this hypothesis: a 1400-fold molar excess of the unlabeled oligonucleotides produced no effects on the ratio of the upper to lower labeled complexes (Fig. 3, lanes 4–9).

### CD Spectroscopy

**Figure 4:**
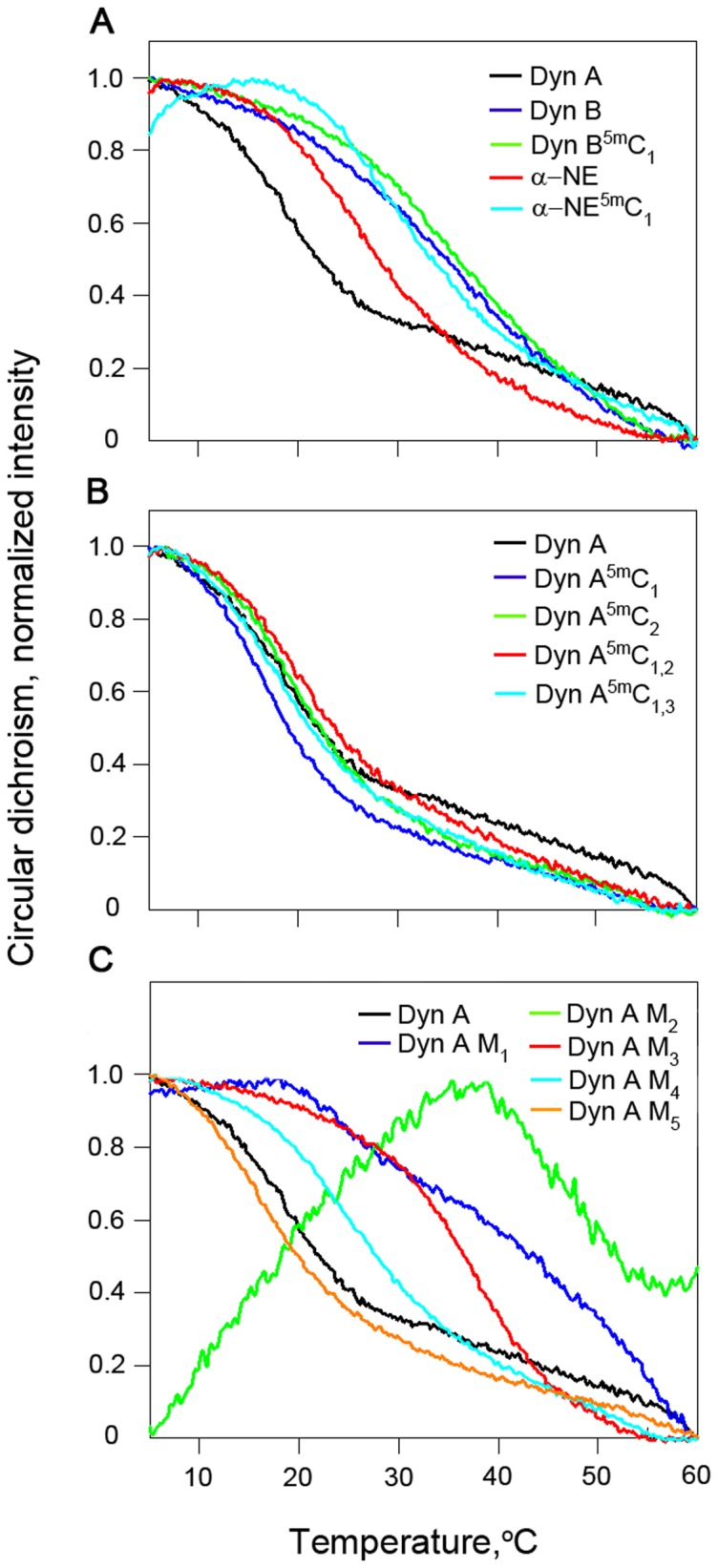
Normalized thermal melting profiles of *PDYN*-derived oligonucleotides recorded with CD spectroscopy (275 nm) between 5°C and 60°C. Normalization was performed using the formula, (S_t_−S_60°C_)/(S_5°C_−S_60°C_), where S_t_, S_5°C_ and S_60°C_ are the signal intensities at 275 nm at a given temperature, 5°C, and 60°C, respectively. A. Black – Dyn A; Blue – Dyn B; Green – Dyn B^5m^C_1_; Red – α-NE; Turquoise – α-NE^5m^C_1_. B. Black – Dyn A; Blue – Dyn A^5m^C_1_; Green – Dyn A^5m^C_2_; Red – Dyn A^5m^C_1,2_; Turquoise – Dyn A^5m^C_1,3_. C. Black – Dyn A; Blue – Dyn A M_1_; Green – Dyn A M_2_; Red – Dyn A M_3_; Turquoise – Dyn A M_4_; Orange - Dyn A M_5_.

To characterize the ssDNA secondary structures and their temperature dependency, CD melting profiles for all *PDYN*-derived oligonucleotides were recorded between 5°C and 60°C at 275 nm, i.e. the wavelength with maximum intensity of the CD signal (Fig. 4), and complete CD spectra were recorded at 4°C and 60°C (Fig. S2). The CD spectra at 5°C were typical for ssDNA not forming DNA G-quartets and triplexes. With exception for Dyn A M_2_, all CD signals decreased when temperature increased from 5°C to 60°C, indicating a loss of secondary structure, although the patterns of signal decrease differed between the samples (Fig. 4). The Dyn A, Dyn A^5m^C_1_, Dyn A^5m^C_2_, Dyn A^5m^C_1,2_, Dyn A^5m^C_1,3_, Dyn A M_4_, Dyn A M_5_ and α-NE oligonucleotides displayed a substantial reduction of the CD signal between 5°C and 30°C (Fig. 4) indicating a) loss of secondary structure, and b) cooperativity of this process that may be due to stabilization of structures mostly by hydrogen bonds but not stacking interactions. In contrast, the Dyn A M_1_, Dyn A M_3_, Dyn B, Dyn B^5m^C_1_ and α-NE^5m^C_1_ oligomers showed higher stabilities with melting temperatures around 35°C.

The M_3_ point mutation increased the melting temperature from approximately 15°C for wild type Dyn A to approximately 35°C. The Dyn A M_1_ oligonucleotide displayed a rather even loss of signal characterized by two transitional intervals at approximately 25°C and 40°C, suggesting two distinct structural conformations differing in melting temperature. Such presence of dual structures could also explain the anomalous behavior of the Dyn A M_2_ oligonucleotide, and of the α-NE^5m^C_1_ oligonucleotide that displayed an initial increase of CD signal with temperature, followed by a decrease after 30–40°C (Fig. 4). The two conformations with different CD signals may exist in equilibrium; when the temperature is increased these oligonucleotides do not melt into unstructured ssDNA, but instead adopt the conformation with stronger CD signal. At the highest temperatures however, all ssDNA structures melt and the standard decrease in CD intensity is observed.

### NMR Spectroscopy

**Figure 5:**
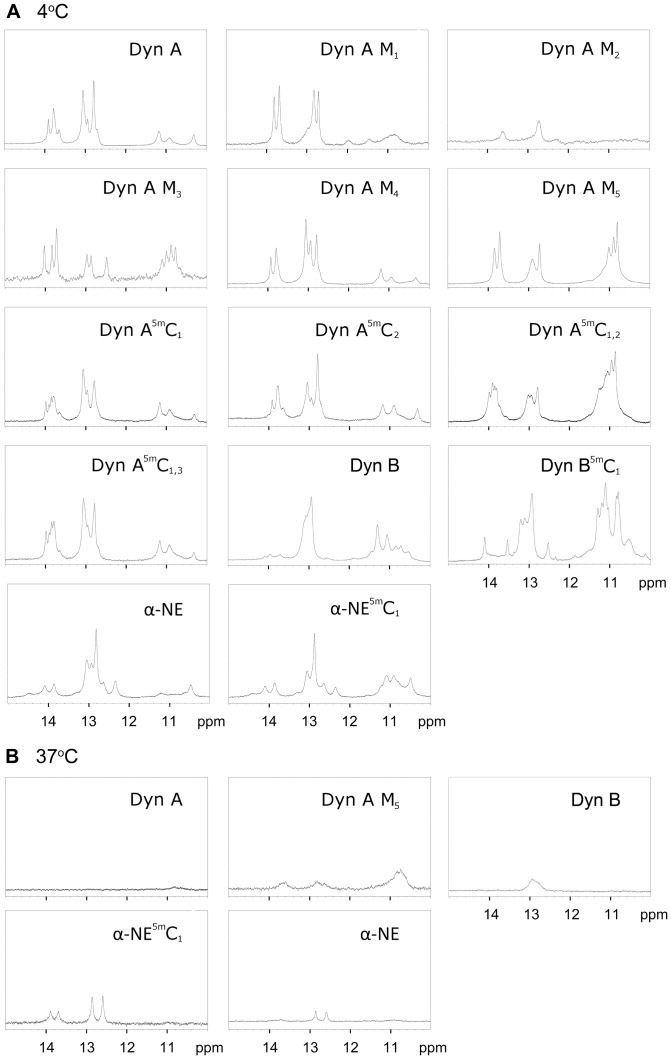
1D ^1^H NMR spectra showing the imino proton region of the PDYN-derived oligonucleotides. Hydrogen-bonded imino protons originating from A-T base pairs (14–15 ppm), G-C base pairs (12–13 ppm) and G-T base pairs (10–12 ppm) are observed. Spectra were recorded at 4°C (**A**) or 37°C (**B**). The oligonucleotide abbreviations used in the figure are presented in Table 1.

1D ^1^H-NMR experiments were performed to monitor the presence of G or T imino proton resonances, which are indicative of DNA hydrogen bonds and consequently base pair formation. At 4°C the *PDYN*-derived oligonucleotides and their methylated and mutated variants display multiple imino proton resonances originating from various base-pair hydrogen bonds (Fig. 5A), confirming the formation of secondary structures involving DNA base-pairs at this temperature. Imino protons were observed from both Watson-Crick A-T base pairs (14–15 ppm) and G-C base pairs (12–13 ppm), as well as from non-canonical G-T pairs (10–12 ppm). The minimum number of identified base pairs of each type is presented in Table 2. Due to spectral overlap, formation of additional base pairs can not be ruled out. The oligonucleotides displayed different patterns of imino proton resonance (Fig. 5, Table 2). For example, the Dyn A-oligonucleotide spectrum at 4°C contains ten imino proton resonances from three A-T pairs, four G-C pairs, and three G-T base pairs (Table 2). The observed hydrogen bonds may derive either from base pairs in a specific secondary structure, or from multiple secondary structures existing in equilibrium. Therefore, the type(s) of secondary structure(s) formed cannot be elucidated from the imino proton data alone.

Methylation of the Dyn A oligonucleotide at one or two CpG sites leads to NMR spectra with eleven (Dyn A^5m^C_1_), nine (Dyn A^5m^C_2_), eleven (Dyn A^5m^C_1,2_) and ten (Dyn A^5m^C_1,3_) imino proton resonances/hydrogen bonds (Table 2). The overall higher number of hydrogen bonds observed in the methylated oligonucleotides may derive from formation of secondary structures with a larger number of base pairs or from the co-existence of multiple secondary structures for each oligonucleotide. The imino proton spectra for Dyn A^5m^C_1_, Dyn A^5m^C_2_ and Dyn A^5m^C_1,3_ are rather similar to the spectrum of unmethylated Dyn A; the main difference appears to be one or two additional A-T base pairs in the methylated oligomers (Fig. 5). The imino proton spectrum of Dyn A^5m^C_1,2_ exhibited larger differences, as it displayed a plethora of resonances originating from G-T base pairs. Also for the α-NE and Dyn B oligonucleotides CpG methylation alters the secondary structure and gives rise to additional base-pair hydrogen bonds (Fig. 5A, Table 2).

Five mutated Dyn A sequences yielded NMR spectra with respectively two (Dyn A M_2_), four (Dyn A M_1_), seven (Dyn A M_5_), eight (Dyn A M_4_) and ten (Dyn A M_3_) imino proton resonances/hydrogen bonds (Table 2). The different hydrogen bond patterns show that these single nucleotide mutations significantly change the base pair patterns, and consequently the secondary structure of the oligonucleotides.

At 37°C wild type and modified Dyn A oligonucleotides displayed no imino proton resonances with the exception of Dyn A M_5_. The loss of imino proton resonances most likely reflects the loss of secondary structure at this temperature. It should however be noted that increased temperature also causes more rapid DNA base-pair opening [45], resulting in faster imino proton exchange accompanied by line broadening. Hence, at elevated temperatures some secondary structures might exist even if imino proton resonances are not observed with NMR spectroscopy. Nevertheless, the presence of a distinct mismatch G-T base pair in Dyn A M_5_ is evidence of base-pairing in this oligonucleotide at higher temperatures. Also α-NE, α-NE^5m^C_1_ and Dyn B retain some residual secondary structure at 37°C (Fig. 5B).

### Computer modeling

**Figure 6:**
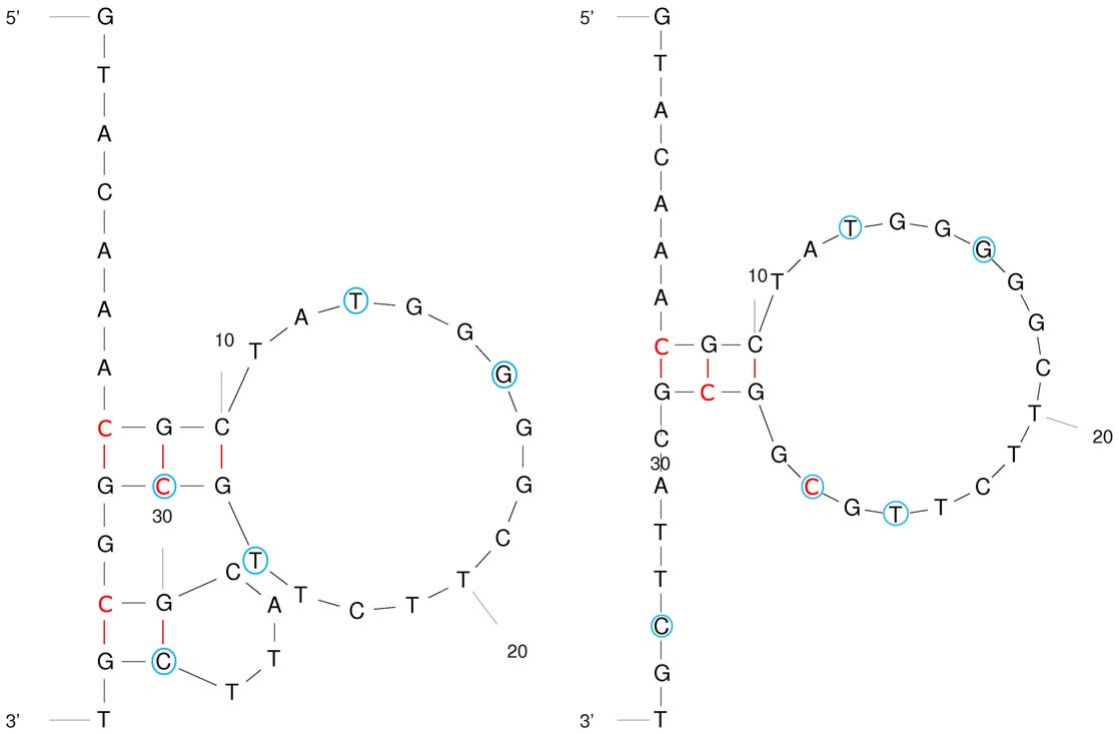
Predicted secondary structures of the Dyn A-coding sequence, calculated with the mFold software (shown as B and C on Fig. S3). Methylated cytosines are shown in red letters, and mutated bases are shown in blue circles.

Secondary structure modeling of the Dyn A and Dyn B sequences with the mFold software yielded a number of different conformations (Figures S3 and S4). Two examples of Dyn A structures are shown in Fig. 6, and the thermodynamic properties of the most favourable secondary structures of the Dyn A- and Dyn B-coding sequences are presented in Tables S2 and S3. The calculated melting temperatures range between 24°C and 42°C for Dyn A, and between 42°C and 54°C for the more stable Dyn B sequences. These results show that for both sequences, a large number of conformations are possible at lower temperatures. As expected, the structures containing non-canonical G-T base pairs, such as Dyn A structures F and G, are only stable at low temperatures.

For the Dyn A sequence, the overall most favourable energetics is exhibited by Dyn A structure B, which consists of one large and one small stem-loop. Dyn A structure A is a variant of structure B, where the smaller stem-loop has melted. Dyn A structure A and C are very similar as they both involve a single loop with a CGC/GCG stem, but the particular bases included in the stem differ between the two structures. In structures A and B bases 8–10 are paired with bases 25–27, while in structure C bases 8–10 and 28–30 are paired together (Fig. 6). Interestingly, although structure B is more stable than structure C at 5 and 31°C, mFold predicts structure C to be more stable at temperatures above 31°C (Table S2). This indicates a fine equilibrium between structures B and C, suggesting that the relative order of stability between these two conformations may vary with factors such as temperature. Fig. 6 shows the positions of the cytosine methylation sites and the mutated bases in Dyn A structures B and C. For structure B, all methylated cytosines are base-paired and located in the two stems, as are two of the five mutated bases. Thus, at least the two mutations, and possibly also the cytosine methylations, are expected to induce conformational changes in this structure. For Dyn A structure C, two methylated cytosines and no mutated bases are base-paired.

The Dyn B structures are generally more stable than the Dyn A structures, mostly because the Dyn B sequence has the capacity to form stable stem-loop structures with long stems. The most stable Dyn B conformation – structure A – has four consecutive G-C base pairs followed by an A-T pair in the stem, giving this structure a melting temperature of 53.8°C. Also for the Dyn B sequence, there is a structure – number C – that is energetically very favourable at low temperatures, but rather unfavourable at higher temperatures (Table S3). This again shows that the equilibrium determining the proportions between the different conformations will vary with the temperature. And again, as the methylated cytosine positions are involved in base-pair formation (Fig. 6), it appears plausible that such methylation may affect the conformational equilibrium as well.

## Discussion

The results obtained from native PAGE, CD and NMR spectroscopy are in general agreement and demonstrate that the *PDYN*-derived oligonucleotides form secondary structures that a) are stabilized by canonical A-T and G-C and non-canonical G-T base pairing, b) display characteristic CD melting profiles, and c) show differential mobility on a native gel. Cytosine methylation appears to induce the formation of novel conformers or shift the equilibrium between existing conformational variants. The effects of CpG methylation are nucleotide sequence dependent, comparable in strength with those induced by point mutations, and are manifested at 4°C but not at 37°C.

PAGE analysis demonstrated that Dyn A, Dyn B and Dyn B (AS) oligonucleotides may adopt compact conformations characterized by higher mobility on a native gel, that are evident at 4°C (Dyn A), or at both 4°C and 37°C (Dyn B and Dyn B (AS)). The α-NE oligomer may be present either in an unstructured form, or in a structured form that does not differ in mobility from those of the reference oligonucleotides at 4°C and 37°C. Methylation of the Dyn B oligonucleotide and of the Dyn A oligomer at the first CpG site (Dyn A^5m^C_1_) substantially decreased the mobility at 4°C or at both temperatures, respectively. In contrast, methylation of the α-NE oligomer and the Dyn A oligonucleotide at the second CpG site (Dyn A^5m^C_2_) did not affect the native gel mobility. Although methylation- and structure-dependent base stacking interactions of the oligonucleotides with the polyacrylamide matrix can not be completely ruled out as a cause for the mobility differences, it appeared more likely that the methylation-induced mobility differences arise from conformational differences between the un-methylated and methylated molecules.

The CD analyses revealed secondary structure formation in the *PDYN*-derived oligonucleotides at low temperatures. When the temperature is increased these structures melt into unstructured or alternative conformations. The pathogenic M_1_–M_3_ mutations had a large impact on the Dyn A oligonucleotide secondary structures and increased their thermostability. In contrast, cytosine methylation did not substantially affect the thermal stabilities of the oligomers. NMR spectroscopy identified canonical A-T and G-C, and non-canonical G-T base-pairing in all *PDYN*-derived oligonucleotides at 4°C. These secondary structures melt upon increased temperature, as expected, although some oligonucleotides retain secondary structure elements also at 37°C. Both the CD and NMR experiments were carried out in an environment containing monovalent ions only, therefore we expect the observed structures to display higher thermal stability *in vivo*, i.e. in an environment containing stabilizing proteins and divalent ions. The NMR, CD, and computer modeling results all showed the presence of multiple secondary structures with different melting temperatures, co-existing in equilibrium. The NMR results at 4°C show that both cytosine methylation and nucleotide mutation affects the base pairing patterns, indicating that both single cytosine methylations and single nucleotide mutations can shift the conformational equilibrium in the ssDNA oligonucleotides. This strongly suggests that the methylated cytosines form base pairs in the observed structures. The mFold structure calculations indicate that the three energetically most favourable Dyn A structures contain a loop-stem involving two methylated cytosines. This loop-stem consists of bases 8–10 (CGC) paired either with bases 25–27 (GCG) in structures A and B, or with bases 28–30 (GCG) in structure C (Fig. 6). Rather similar free energies are obtained by base-pairing with either the 25–27 GCG or the 28–30 GCG sequence, and it is conspicuous that both central cytosines, C26 and C29, constitute methylation sites. It is plausible that methylation of C26 and/or C29 can affect the preference of bases 8–10 to form pairs with the 25–27 or the 28–30 GCG triad. Hence, even though the actual secondary structures cannot be elucidated from current experiments, and even though the current computational tools for structure prediction of ssDNA can not evaluate impact of cytosine methylation, the mFold results together with spectroscopic data make it likely that slightly different base-pairing properties of 5-methylcytosine compared to normal cytosine influences the secondary structure formation of ssDNA. Density functional theory (DFT) calculations have demonstrated improved stacking energies for 5-methylcytosine [46], which might make a loop-stem with 5-methylcytosine energetically more favourable than a loop-stem with unmethylated cytosine.

Such a methylation effect is particularly evident for the Dyn B sequence, where the NMR results show an increased number of G-C and G-T base pairs as a result of 5mC1-methylation (Fig. 5). In total 14 imino proton resonances corresponding to Watson-Crick base pairs are observed, and it appears but impossible to observe so many base pairs in one structural conformation of the Dyn B sequence. Thus, the increased number of observed base pairs indicates that ^5m^C_1_-methylation has caused an increase in the number of stable conformations (at 4°C). The existence of multiple conformations in the Dyn B^5m^C_1_ NMR sample provides a simple explanation for the two bands observed on the PAGE gel for the same sequence (Fig. 2, Table 2).

CpG sites in the dynorphin-coding segment of the *PDYN* gene are differentially methylated in human tissues [47]. Methylation of the first CpG site in the Dyn A sequence, but not of other sites in the CpG-rich segment covering the α-NE and Dyn sequences, is conserved between human subjects, but strongly differs between brain and peripheral human tissues, and also between tissues and cultured human cell lines. This site is furthermore located within a mutational “hot spot” and is destroyed by two pathogenic missense mutations that induce human neurodegenerative disorder SCA23 ([35], manuscript in preparation). Taken together, these observations suggest that methylation of this CpG dinucleotide has epigenetic regulatory consequences, possibly caused by the ssDNA secondary structure effects that we observe with PAGE and NMR analysis.

Formation of non-canonical ssDNA structures may affect DNA-protein and DNA-DNA interactions, and consequently stimulate or repress transcription, DNA repair, recombination or replication [1]–[5]. Such structures may be formed by ssDNA under conditions of DNA supercoiling that affects gene regulation in pro- and eukaryotes [48]–[50]. One example is the p53 tumor repressor protein which displays stronger binding to its DNA target site when the latter adopts a stem-loop conformation [51]. Our data indicate that cytosine methylation affects the conformational flexibility of short ssDNA molecules and their propensity to form secondary structures, both at 4°C and 37°C. Given these results, it becomes important to examine whether cytosine methylation may interfere with the conformational flexibility of ssDNA segments in vivo in a chromatin context under conditions of a crowded intranuclear environment and DNA superhelical stress. Such effects may be relevant for the processes with which double-stranded DNA unwinds to ssDNA during gene transcription, DNA replication, and recombination.

## Supporting Information

Figure S1 **Mass spectrum for (A) Dyn A and (B) Dyn A^5m^C_1_ oligonucleotides.**
(TIF)

Figure S2 **CD spectra between 220 and 340 nm for selected Dyn oligonucleotides in 10 mM sodium phosphate buffer, pH 7.3, at 4°C (solid lines) and at 60°C (dashed lines). The oligonucleotide abbreviations used in the figure are presented in** **Table 1** **.**
(TIF)

Figure S3 **Seven Dyn A secondary structures, which thermodynamic properties were calculated using mFold software** [**36**] **and listed in Table S2.**
(TIF)

Figure S4 **Six Dyn B secondary structures, which thermodynamic properties were calculated using the mFold software** [**36**] **and listed in Table S3.**
(TIF)

Table S1 **Mass of oligonucleotides used in the study determined by mass spectrometry.**
(DOCX)

Table S2 **Melting temperature (Tm), number of G-C, A-T, and G-T base pairs, and folding energies (ΔG) between 5 and 37°C for seven different secondary structures of the Dyn A-coding sequence.**
(DOC)

Table S3 **Melting temperature (Tm), number of G-C, A-T, and G-T base pairs, and folding energies (ΔG) between 5 and 55°C for seven different secondary structures of the Dyn B-coding sequence.**
(DOC)

## References

[1] Chen Z, Yang H, Pavletich NP (2008). Mechanism of homologous recombination from the RecA-ssDNA/dsDNA structures.. Nature.

[2] Swamynathan SK, Nambiar A, Guntaka RV (1998). Role of single-stranded DNA regions and Y-box proteins in transcriptional regulation of viral and cellular genes.. FASEB J.

[3] Masai H, Arai K (1997). Frpo: a novel single-stranded DNA promoter for transcription and for primer RNA synthesis of DNA replication.. Cell.

[4] Zou L, Elledge SJ (2003). Sensing DNA damage through ATRIP recognition of RPA-ssDNA complexes.. Science.

[5] Jackson SP (2002). Sensing and repairing DNA double-strand breaks.. Carcinogenesis.

[6] Patel SS, Pandey M, Nandakumar D (2011). Dynamic coupling between the motors of DNA replication: hexameric helicase, DNA polymerase, and primase.. Curr Opin Chem Biol.

[7] Siddiqui-Jain A, Grand CL, Bearss DJ, Hurley LH (2002). Direct evidence for a G-quadruplex in a promoter region and its targeting with a small molecule to repress c-MYC transcription.. Proc Natl Acad Sci U S A.

[8] Sakamoto N, Ohshima K, Montermini L, Pandolfo M, Wells RD (2001). Sticky DNA, a self-associated complex formed at long GAA*TTC repeats in intron 1 of the frataxin gene, inhibits transcription.. J Biol Chem.

[9] Pearson CE, Zorbas H, Price GB, Zannis-Hadjopoulos M (1996). Inverted repeats, stem-loops, and cruciforms: significance for initiation of DNA replication.. J Cell Biochem.

[10] Faruqi AF, Datta HJ, Carroll D, Seidman MM, Glazer PM (2000). Triple-helix formation induces recombination in mammalian cells via a nucleotide excision repair-dependent pathway.. Mol Cell Biol.

[11] Napierala M, Parniewski P, Pluciennik A, Wells RD (2002). Long CTG.CAG repeat sequences markedly stimulate intramolecular recombination.. J Biol Chem.

[12] Wells RD (2007). Non-B DNA conformations, mutagenesis and disease.. Trends Biochem Sci.

[13] Lada AG, Waisertreiger IS, Grabow CE, Prakash A, Borgstahl GE (2011). Replication protein A (RPA) hampers the processive action of APOBEC3G cytosine deaminase on single-stranded DNA.. PLoS One.

[14] Biyani M, Nishigaki K (2005). Single-strand conformation polymorphism (SSCP) of oligodeoxyribonucleotides: an insight into solution structural dynamics of DNAs provided by gel electrophoresis and molecular dynamics simulations.. J Biochem.

[15] Liang X, Kuhn H, Frank-Kamenetskii MD (2006). Monitoring single-stranded DNA secondary structure formation by determining the topological state of DNA catenanes.. Biophys J.

[16] Orita M, Iwahana H, Kanazawa H, Hayashi K, Sekiya T (1989). Detection of polymorphisms of human DNA by gel electrophoresis as single-strand conformation polymorphisms.. Proc Natl Acad Sci U S A.

[17] Tomasko M, Vorlickova M, Sagi J (2009). Substitution of adenine for guanine in the quadruplex-forming human telomere DNA sequence G(3)(T(2)AG(3))(3).. Biochimie.

[18] Bernstein BE, Meissner A, Lander ES (2007). The mammalian epigenome.. Cell.

[19] Maunakea AK, Nagarajan RP, Bilenky M, Ballinger TJ, D'Souza C (2010). Conserved role of intragenic DNA methylation in regulating alternative promoters.. Nature.

[20] Deaton AM, Bird A (2011). CpG islands and the regulation of transcription.. Genes Dev.

[21] Bird A (2002). DNA methylation patterns and epigenetic memory.. Genes Dev.

[22] Ballestar E, Wolffe AP (2001). Methyl-CpG-binding proteins. Targeting specific gene repression.. Eur J Biochem.

[23] Straussman R, Nejman D, Roberts D, Steinfeld I, Blum B (2009). Developmental programming of CpG island methylation profiles in the human genome.. Nat Struct Mol Biol.

[24] Mayer-Jung C, Moras D, Timsit Y (1998). Hydration and recognition of methylated CpG steps in DNA.. EMBO J.

[25] Hodges-Garcia Y, Hagerman PJ (1992). Cytosine methylation can induce local distortions in the structure of duplex DNA.. Biochemistry.

[26] Zacharias W, Jaworski A, Wells RD (1990). Cytosine methylation enhances Z-DNA formation in vivo.. J Bacteriol.

[27] Severin PM, Zou X, Gaub HE, Schulten K (2011). Cytosine methylation alters DNA mechanical properties.. Nucleic Acids Res.

[28] Geahigan KB, Meints GA, Hatcher ME, Orban J, Drobny GP (2000). The dynamic impact of CpG methylation in DNA.. Biochemistry.

[29] Derreumaux S, Chaoui M, Tevanian G, Fermandjian S (2001). Impact of CpG methylation on structure, dynamics and solvation of cAMP DNA responsive element.. Nucleic Acids Res.

[30] Meints GA, Drobny GP (2001). Dynamic impact of methylation at the M. Hhai target site: a solid-state deuterium NMR study.. Biochemistry.

[31] Nathan D, Crothers DM (2002). Bending and flexibility of methylated and unmethylated EcoRI DNA.. J Mol Biol.

[32] Mergny JL, Lacroix L (2003). Analysis of thermal melting curves.. Oligonucleotides.

[33] Vondruskova J, Kypr J, Kejnovska I, Fialova M, Vorlickova M (2008). Role of loops in the guanine quadruplex formation by DNA/RNA hybrid analogs of G4T4G4.. Int J Biol Macromol.

[34] Skolakova P, Bednarova K, Vorlickova M, Sagi J (2010). Quadruplexes of human telomere dG(3)(TTAG(3))(3) sequences containing guanine abasic sites.. Biochem Biophys Res Commun.

[35] Bakalkin G, Watanabe H, Jezierska J, Depoorter C, Verschuuren-Bemelmans C (2010). Prodynorphin mutations cause the neurodegenerative disorder spinocerebellar ataxia type 23.. Am J Hum Genet.

[36] Zuker M (2003). Mfold web server for nucleic acid folding and hybridization prediction.. Nucleic Acids Res.

[37] Nishigaki K, Husimi Y, Tsubota M (1986). Detection of differences in higher order structure between highly homologous single-stranded DNAs by low-temperature denaturant gradient gel electrophoresis.. J Biochem.

[38] Diekmann S, Lilley DM (1987). The anomalous gel migration of a stable cruciform: temperature and salt dependence, and some comparisons with curved DNA.. Nucleic Acids Res.

[39] Han H, Langley DR, Rangan A, Hurley LH (2001). Selective interactions of cationic porphyrins with G-quadruplex structures.. J Am Chem Soc.

[40] Laporte L, Thomas GJ (1998). A hairpin conformation for the 3′ overhang of Oxytricha nova telomeric DNA.. J Mol Biol.

[41] Owen BA, Yang Z, Lai M, Gajec M, Badger JD (2005). (CAG)(n)-hairpin DNA binds to Msh2-Msh3 and changes properties of mismatch recognition.. Nat Struct Mol Biol.

[42] Shea RG, Ng P, Bischofberger N (1990). Thermal denaturation profiles and gel mobility shift analysis of oligodeoxynucleotide triplexes.. Nucleic Acids Res.

[43] Yu A, Dill J, Wirth SS, Huang G, Lee VH (1995). The trinucleotide repeat sequence d(GTC)15 adopts a hairpin conformation.. Nucleic Acids Res.

[44] Zhuang XY, Tang J, Hao YH, Tan Z (2007). Fast detection of quadruplex structure in DNA by the intrinsic fluorescence of a single-stranded DNA binding protein.. J Mol Recognit.

[45] Warmlander S, Sen A, Leijon M (2000). Imino proton exchange in DNA catalyzed by ammonia and trimethylamine: evidence for a secondary long-lived open state of the base pair.. Biochemistry.

[46] Acosta-Silva C, Branchadell V, Bertran J, Oliva A (2010). Mutual relationship between stacking and hydrogen bonding in DNA. Theoretical study of guanine-cytosine, guanine-5-methylcytosine, and their dimers.. J Phys Chem B.

[47] Bakalkin G, Kononenko O, Taqi M, Watanabe H, Krishtal O (2010). Methylation of the enkephalin-encoding sequences in the human prodynorphin gene: Specific patterns in brain and peripheral tissues. Program No. 167.16. 2010 Neuroscience Meeting Planner.

[48] Wright BE, Schmidt KH, Hunt AT, Lodmell JS, Minnick MF (2011). The roles of transcription and genotoxins underlying p53 mutagenesis in vivo.. Carcinogenesis.

[49] Krasilnikov AS, Podtelezhnikov A, Vologodskii A, Mirkin SM (1999). Large-scale effects of transcriptional DNA supercoiling in vivo.. J Mol Biol.

[50] Hernandez M, Wright SD, Cai TQ (2007). Critical role of cholesterol ester transfer protein in nicotinic acid-mediated HDL elevation in mice.. Biochem Biophys Res Commun.

[51] Gohler T, Reimann M, Cherny D, Walter K, Warnecke G (2002). Specific interaction of p53 with target binding sites is determined by DNA conformation and is regulated by the C-terminal domain.. J Biol Chem.

