# Supplementary figures and images for: Conformation Effects of CpG Methylation on Single-Stranded DNA Oligonucleotides: Analysis of the Opioid Peptide Dynorphin-Coding Sequences
Source: PLoS One. 2012 Jun 29;7(6):e39605. doi: 10.1371/journal.pone.0039605 (PMC3387154; doi:10.1371/journal.pone.0039605)

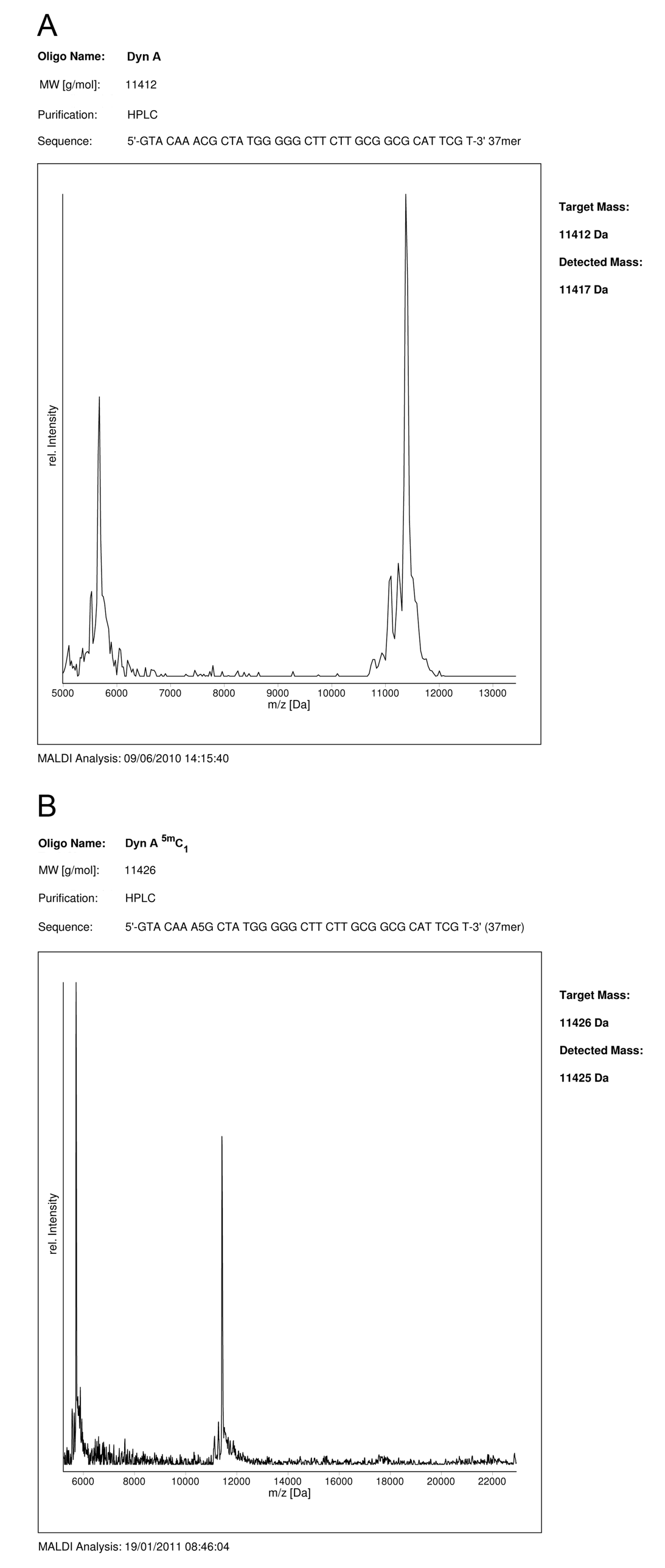

Supplement: Figure S1 — Mass spectrum for (A) Dyn A and (B) Dyn A5mC1 oligonucleotides. (TIF) [file pone.0039605.s001.tif]

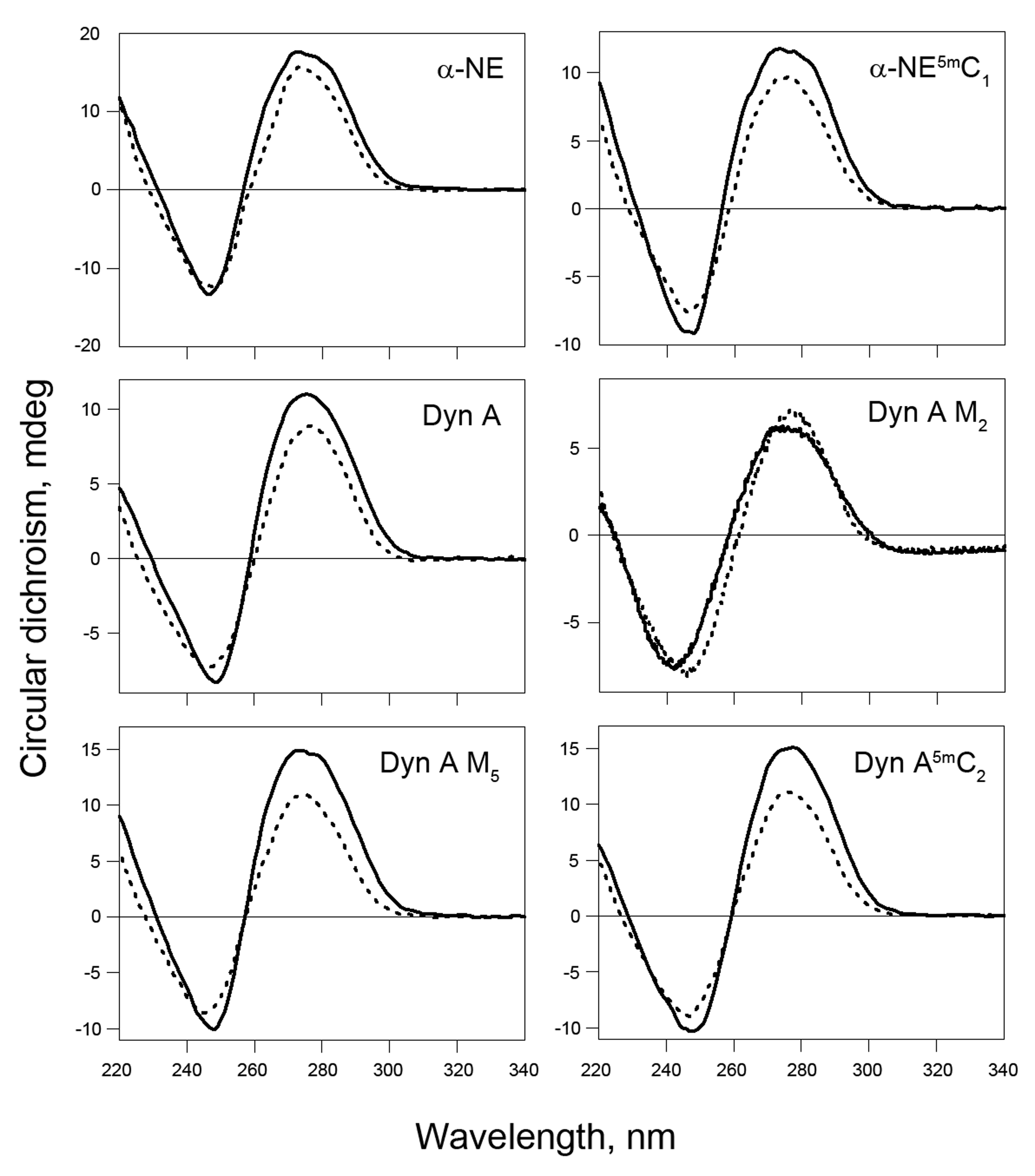

Supplement: Figure S2 — CD spectra between 220 and 340 nm for selected Dyn oligonucleotides in 10 mM sodium phosphate buffer, pH 7.3, at 4°C (solid lines) and at 60°C (dashed lines). The oligonucleotide abbreviations used in the figure are presented in Table 1 . (TIF) [file pone.0039605.s002.tif]

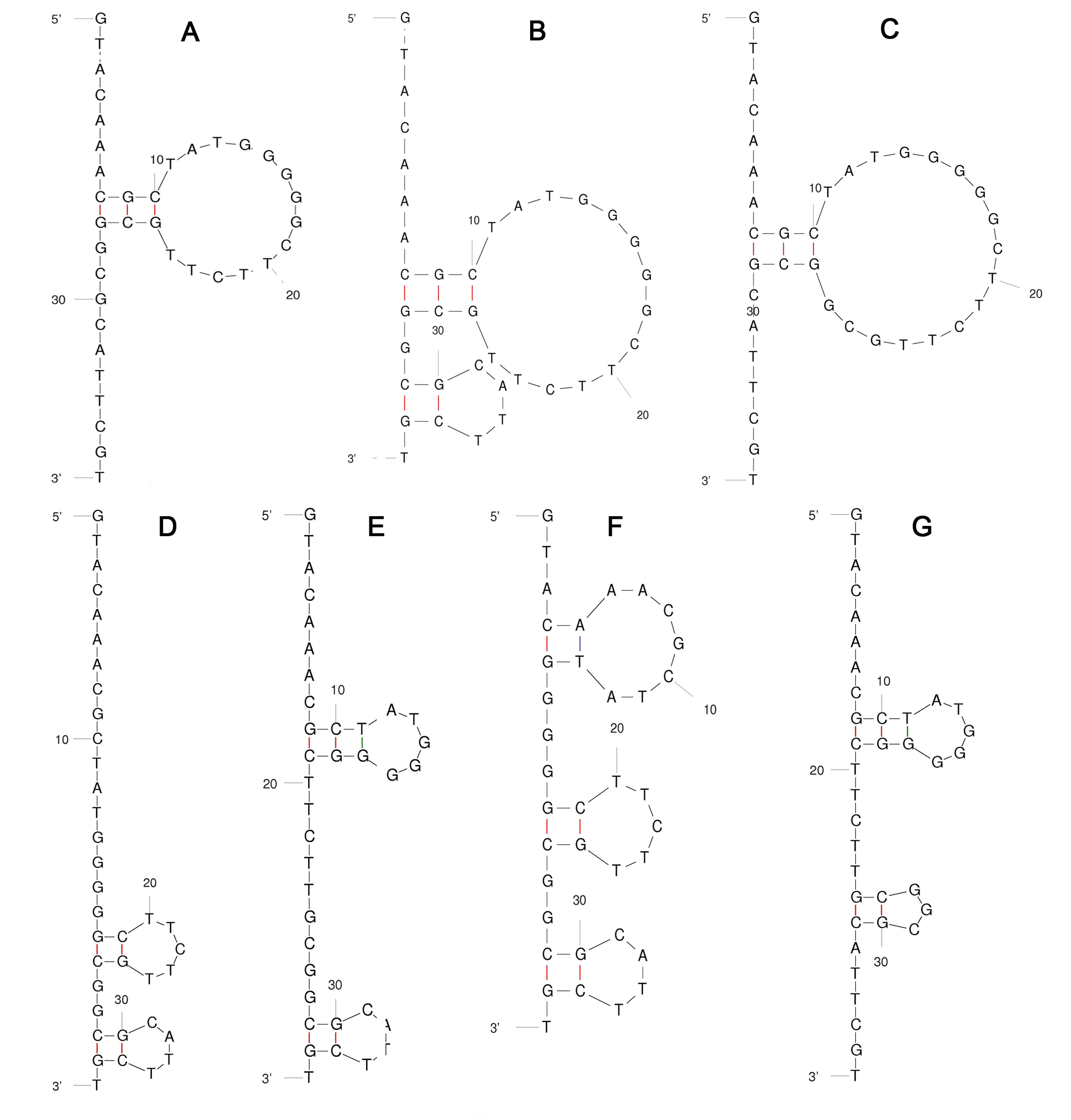

Supplement: Figure S3 — Seven Dyn A secondary structures, which thermodynamic properties were calculated using mFold software [36] and listed in Table S2. (TIF) [file pone.0039605.s003.tif]

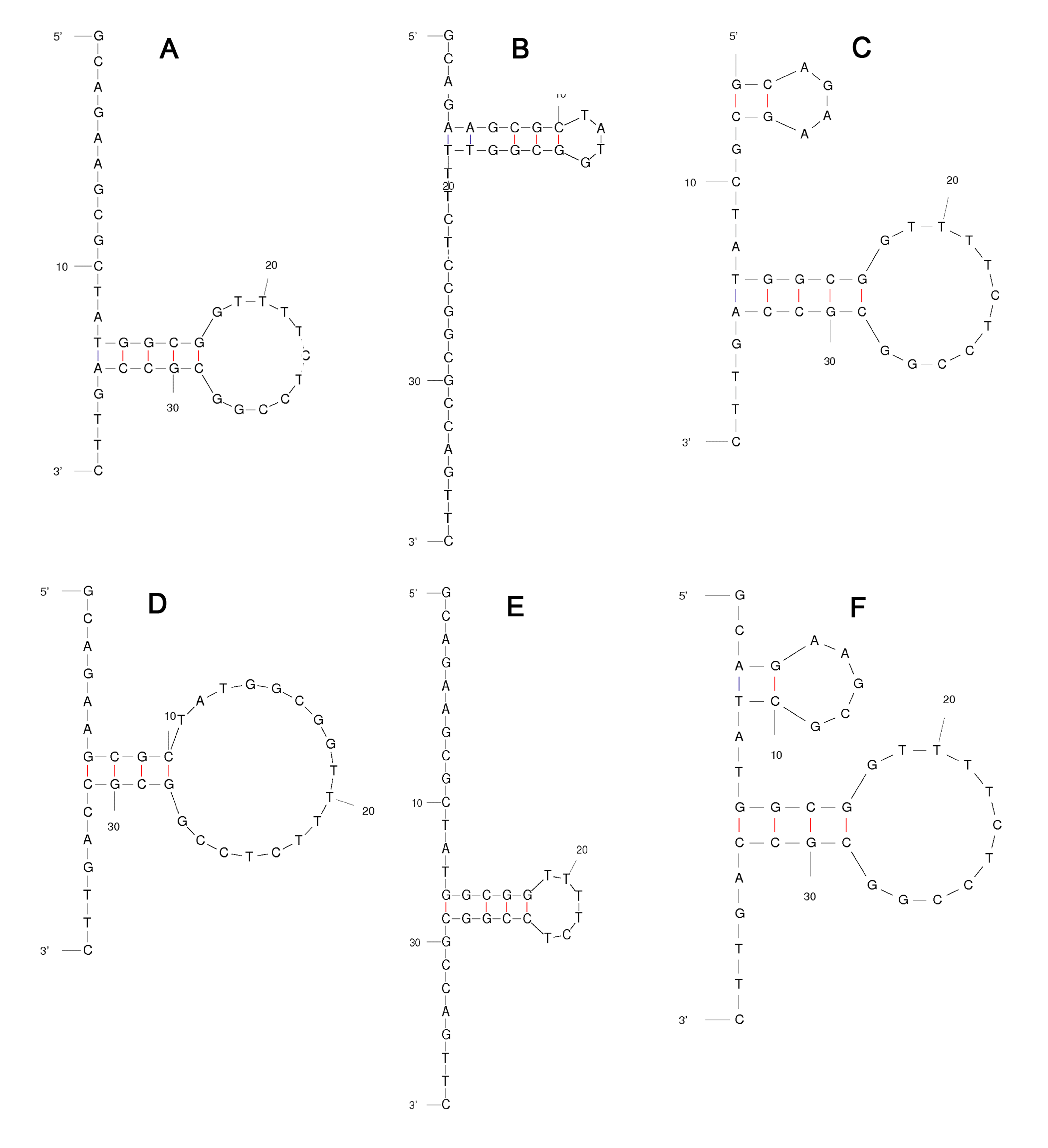

Supplement: Figure S4 — Six Dyn B secondary structures, which thermodynamic properties were calculated using the mFold software [36] and listed in Table S3. (TIF) [file pone.0039605.s004.tif]
