# Supplementary material for: Conformation Effects of CpG Methylation on Single-Stranded DNA Oligonucleotides: Analysis of the Opioid Peptide Dynorphin-Coding Sequences
Source: PLoS One. 2012 Jun 29;7(6):e39605. doi: 10.1371/journal.pone.0039605 (PMC3387154; doi:10.1371/journal.pone.0039605)
Supplement: Table S1 — Mass of oligonucleotides used in the study determined by mass spectrometry. (DOCX) [file pone.0039605.s004a.tif]

**Table S1.** Mass of oligonucleotides used in the study determined by mass spectrometry

| Oligonucleotide | Expected mass in Da | Observed mass in Da | Deviation from expected mass (%) |
| --- | --- | --- | --- |
| α-NE | 11389 | 11386 | -0.03 |
| α-NE^5m^C_1_ | 11403 | 11401 | -0.02 |
| Dyn A | 11412 | 11417 | 0.05 |
| Dyn A^5m^C_1_ | 11426 | 11425 | -0.01 |
| Dyn A^5m^C_2_ | 11426 | 11430 | 0.03 |
| Dyn A^5m^C_1,2_ | 11440 | 11438 | -0.02 |
| Dyn A^5m^C_1,3_ | 11440 | 11444 | 0.03 |
| Dyn A M_1_ | 11426 | 11430 | 0.03 |
| Dyn A M_2_ | 11427 | 11433 | 0.05 |
| Dyn A M_3_ | 11427 | 11427 | 0 |
| Dyn A M_4_ | 11372 | 11372 | 0 |
| Dyn A M_5_ | 11421 | 11417 | -0.04 |
| Dyn A (AS) | 11328 | 11328 | 0 |

The MALDI instrument used, had a measuring accuracy of +/- 0.3%; the deviation from expected mass for each oligonucleotide used in this study are within the instrument measuring accuracy range.
