# Supplementary material for: Conformation Effects of CpG Methylation on Single-Stranded DNA Oligonucleotides: Analysis of the Opioid Peptide Dynorphin-Coding Sequences
Source: PLoS One. 2012 Jun 29;7(6):e39605. doi: 10.1371/journal.pone.0039605 (PMC3387154; doi:10.1371/journal.pone.0039605)
Supplement: Table S2 — Melting temperature (Tm), number of G-C, A-T, and G-T base pairs, and folding energies (ΔG) between 5 and 37°C for seven different secondary structures of the Dyn A-coding sequence. (DOC) [file pone.0039605.s005.doc]

**Table S2.** Melting temperature (Tm), number of G-C, A-T, and G-T base pairs, and folding energies (ΔG) between 5 and 37 °C for seven different secondary structures of the Dyn A-coding sequence

|  | **Secondary structure** | | | | | | |
| --- | --- | --- | --- | --- | --- | --- | --- |
|  | **A** | **B** | **C** | **D** | **E** | **F** | **G** |
| **T_m_** | 41.8 °C | 35.5 °C | 37.3 °C | 29.1 °C | 26.6 °C | 23.8 °C | 24.0 °C |
| **G-C** | 3 | 5 | 3 | 4 | 4 | 5 | 4 |
| **A-T** | 0 | 0 | 0 | 0 | 0 | 1 | 0 |
| **G-T** | 0 | 0 | 0 | 0 | 1 | 0 | 1 |
| **Folding energy** **ΔG, kcal/mol** | | | | | | | |
| **37 °C** | -0.55 | 0.23 | -0.07 |  |  |  |  |
| **35 °C** | -0.76 | -0.11 | -0.26 |  |  |  |  |
| **33 °C** | -1 | -0.47 | -0.47 |  |  |  |  |
| **31 °C** | -1.23 | -0.84 | -0.69 |  |  |  |  |
| **29 °C** | -1.44 | -1.19 | -0.9 | -0.13 |  |  |  |
| **27 °C** | -1.68 | -1.54 | -1.11 | -0.4 |  |  |  |
| **25 °C** |  | -1.9 | -1.31 | -0.69 | -0.25 |  |  |
| **23 °C** |  | -2.27 | -1.53 | -0.97 | -0.55 | -0.24 | -0.16 |
| **21 °C** |  | -2.63 | -1.74 | -1.24 | -0.84 | -0.67 | -0.43 |
| **19 °C** |  | -2.97 | -1.93 | -1.52 | -1.14 | -1.09 | -0.72 |
| **17 °C** |  | -3.35 | -2.16 | -1.81 | -1.47 | -1.54 | -1.03 |
| **15 °C** |  | -3.69 | -2.36 | -2.07 | -1.75 | -1.95 | -1.31 |
| **13 °C** |  | -4.06 | -2.58 |  | -2.06 | -2.41 | -1.59 |
| **11 °C** |  | -4.43 | -2.79 |  | -2.37 | -2.88 |  |
| **9 °C** |  | -4.77 | -2.98 |  | -2.65 | -3.32 | -2.15 |
| **7 °C** |  | -5.13 | -3.2 |  | -2.97 | -3.79 | -2.46 |
| **5 °C** |  | -5.49 | -3.43 |  | -3.27 | -4.23 | -2.74 |

Modeling was carried out using the mFold software [36] using an ionic strength of 10 mM NaCl.
