# Supplementary material for: Conformation Effects of CpG Methylation on Single-Stranded DNA Oligonucleotides: Analysis of the Opioid Peptide Dynorphin-Coding Sequences
Source: PLoS One. 2012 Jun 29;7(6):e39605. doi: 10.1371/journal.pone.0039605 (PMC3387154; doi:10.1371/journal.pone.0039605)
Supplement: Table S3 — Melting temperature (Tm), number of G-C, A-T, and G-T base pairs, and folding energies (ΔG) between 5 and 55°C for seven different secondary structures of the Dyn B-coding sequence. (DOC) [file pone.0039605.s006.doc]

**Table S3.** Melting temperature (Tm), number of G-C, A-T, and G-T base pairs, and folding energies (ΔG) between 5 and 55 °C for seven different secondary structures of the Dyn B-coding sequence

|  | **Secondary structure** | | | | | |
| --- | --- | --- | --- | --- | --- | --- |
|  | **A** | **B** | **C** | **D** | **E** | **F** |
| **T_m_** | 53.8 °C | 50.5 °C | 45.3 °C | 48.1 °C | 46.8 °C | 41.6 °C |
| **G-C** | 4 | 3 | 6 | 4 | 4 | 5 |
| **A-T** | 1 | 2 | 1 | 0 | 0 | 1 |
| **G-T** | 0 | 0 | 0 | 0 | 0 | 0 |
| **Folding energy** **ΔG, kcal/mol** | | | | | | |
| **55 °C** | -0.04 | 0.45 |  | 0.82 |  |  |
| **53 °C** | -0.29 | 0.21 |  | 0.59 |  |  |
| **51 °C** | -0.56 | -0.03 |  | 0.35 |  |  |
| **49 °C** | -0.81 | -0.28 |  | 0.09 |  |  |
| **47 °C** | -1.08 | -0.53 |  | -0.15 |  |  |
| **45 °C** | -1.35 | -0.77 | -0.22 | -0.4 | -0.32 |  |
| **43 °C** | -1.61 | -1.01 | -0.57 | -0.65 | -0.59 |  |
| **41 °C** | -1.86 | -1.28 | -0.9 | -0.91 | -0.85 |  |
| **39 °C** | -2.12 | -1.52 | -1.25 | -1.18 | -1.12 | -0.41 |
| **37 °C** | -2.41 | -1.78 | -1.63 | -1.46 | -1.39 | -0.79 |
| **35 °C** | -2.65 | -2 | -1.95 | -1.7 | -1.64 | -1.11 |
| **33 °C** | -2.91 | -2.26 | -2.3 | -1.96 | -1.91 | -1.45 |
| **31 °C** | -3.17 | -2.53 | -2.64 | -2.23 | -2.17 | -1.78 |
| **29 °C** | -3.43 | -2.8 | -2.99 | -2.49 | -2.43 | -2.13 |
| **27 °C** | -3.69 | -3.05 | -3.34 | -2.77 | -2.69 | -2.45 |
| **25 °C** | -3.96 | -3.33 | -3.7 | -3.03 | -2.95 | -2.81 |
| **23 °C** | -4.22 | -3.59 | -4.04 | -3.29 | -3.22 | -3.14 |
| **21 °C** | -4.48 | -3.86 | -4.38 | -3.55 | -3.49 | -3.48 |
| **19 °C** | -4.73 | -4.11 | -4.71 | -3.8 | -3.74 | -3.82 |
| **17 °C** |  | -4.38 | -5.12 |  |  | -4.17 |
| **15 °C** |  | -4.64 | -5.5 |  |  | -4.5 |
| **13 °C** |  | -4.91 | -5.85 |  |  | -4.84 |
| **11 °C** |  | -5.18 | -6.27 |  |  | -5.21 |
| **9 °C** |  | -5.44 | -6.67 |  |  |  |
| **7 °C** |  | -5.7 | -7.08 |  |  |  |
| **5 °C** |  | -5.97 | -7.46 |  |  |  |

Modeling was carried out using the mFold software [35] using an ionic strength of 10 mM NaCl.
